# Region- and season-specific exposure to lead in a common North American songbird

**DOI:** 10.1007/s10646-026-03070-3

**Published:** 2026-03-27

**Authors:** M. L. Ohrberg, D. J. Becker, G. M. Filippelli, E. D. Ketterson, S. T. Rader, S. M. Reed, E. J. Williams, A. E. Jahn

**Affiliations:** 1 Department of Earth and Environmental Sciences, Indiana University Indianapolis, Indianapolis, IN USA; 2 School of Biological Sciences, University of Oklahoma, Norman, OK USA; 3 Environmental Resilience Institute, Indiana University, Bloomington, IN USA; 4 Department of Biology, Indiana University, Bloomington, IN USA; 5 Department of Earth and Atmospheric Sciences, Indiana University, Bloomington, IN USA; 6 Department of Biology, Georgetown University, Washington, DC USA; 7 Department of Integrative Biology, Oregon State University, Corvallis, OR USA

**Keywords:** Alaska, American Robin, Indiana, heavy metal, Pb

## Abstract

Although birds have been used globally as bioindicators of heavy metals, the determinants and physiological consequences of their exposure to heavy metals remain poorly understood. The American Robin (*Turdus migratorius*) is a common songbird in a variety of habitats across most of North America that could serve as an ideal sentinel of heavy metal contamination. We evaluated how robin exposure to lead (Pb) varied regionally, seasonally and among demographic groups, and assessed impacts on body condition and reproductive hormone concentrations. We detected higher blood Pb concentrations in robins sampled in a more urban region (Indiana) compared to a more rural region (Alaska). Higher blood Pb concentrations in the more urban region may reflect the robin diet of earthworms, which accumulate lead due to their feeding behavior and could incorporate greater ambient soil lead contamination typical of more urban areas. Blood Pb concentrations were also highest in spring and summer, when robins primarily forage on earthworms. We found no relationship between blood Pb concentrations and body condition, but blood Pb concentrations showed a negative relationship with testosterone in male robins and with estradiol in female robins, suggesting lead may have negative effects on reproductive function. Overall, these results suggest that robins could serve as reliable bioindicators of lead and its impacts on wildlife health across a broad range of ecosystems.

**Supplementary Information:**

The online version contains supplementary material available at 10.1007/s10646-026-03070-3.

## Introduction

Lead (Pb) is an anthropogenically concentrated heavy metal that has been used in various industries, resulting in contaminated ecosystems (e.g., Filippelli et al. 2018, 2024). This is especially true of urban areas due to historic use of leaded gasoline in automobiles and Pb paint use (Obeng-Gyasi et al. 2021). Pb exists in both inorganic and organic forms, but the former makes up much more of the environmental burden (Pattee and Pain 2002). Pb bioavailability is primarily controlled by soil properties such as pH, cation exchange capacity, and dissolved organic carbon, so it can be difficult to predict bioavailability and toxicity (Wijayawardena et al. 2017).

Exposure to heavy metals poses various health-related risks across wildlife species (reviewed by Verma et al. 2023). However, the causes and consequences of heavy metal exposure in songbirds, the largest group of birds on the planet (Order Passeriformes), remains poorly understood. Previous studies on birds as bioindicators of heavy metals (reviewed by Celik et al. 2021) have focused on acute heavy metal toxicity or toxicity in association with mining (e.g., Beyer et al. 2004; Hansen et al. 2011) and ammunition and fishing tackle (e.g., Haig et al. 2014), with ammunition and fishing tackle both being well established as Pb hazards from accidental ingestion (Gorski et al. 2021).

Pb toxicity in birds, much like in humans, impacts all body systems and manifests in clinical signs such as anemia, lethargy, and muscle loss, with sublethal effects at blood Pb concentrations as low as 2.5 µg/dL (Franson and Pain 2011). Delta-aminolevulinic acid dehydratase (ALAD), the most Pb-sensitive enzyme and first marker of exposure, is important in the production of heme, which is essential for the regulation of hemoglobin content in red blood cells (Astrin et al. 1987; van den Heever et al. 2024). For example, a study conducted along the Coeur d’Alene River in Idaho, USA found that American robins (*Turdus migratorius*) were exposed to lead levels sufficient to inhibit ALAD by > 50% (Johnson et al. 1999). In the Tri-State Mining District of the US midwest, mean ALAD activity decreased by > 50% in red blood cells of sampled songbirds, including American robins, demonstrating that Pb can have notable impacts on avian health, even at relatively low blood concentrations (Beyer et al. 2004).

American robins are ecologically significant as a bioindicator species because of their wide geographic range across North America and their ability to thrive in natural and anthropogenic habitats (Vanderhoff et al. 2020). Additionally, their diet is broad - predominantly consisting of earthworms and insects in spring, then shifting to berries in fall and winter (Vanderhoff et al. 2020). They are also important in seed dispersal and insect control, and serve as an important food source for larger predators (Gochfeld and Burger 1984; Vanderhoff et al. 2020). The consumption of earthworms in particular makes robins ideal bioindicators of soil Pb contamination (Bennett et al. 2007; Zahor et al. 2024), as the incidental ingestion of soil in earthworms exposes them to this heavy metal (Beyer and Sample 2016). Earthworms are also the main source of heavy metals such as cadmium, mercury, and zinc in robin diets (Beyer and Sample 2016), and robins are known to uptake soil Pb at a higher rate than other songbirds with which they are sympatric (Sample et al. 2011). Bioavailability to earthworms is controlled heavily by soil properties, and acidic soils are highly lethal to earthworms at lower Pb concentrations (Wijayawardena et al. 2017). Concentrations as high as 766 mg/kg dry weight have been detected in earthworm species (*Eisenoides lonnbergi*) collected from soil containing only 17 mg/kg of Pb (Beyer et al. 2018), showing that bioaccumulation can occur at high rates.

To improve our understanding of the potential impacts of exposure to Pb in songbirds, we evaluated the demographic-, season-, and geographic-specific exposure of American robins to Pb, and the potential consequences of Pb on their physiology. Specifically, we compared blood Pb concentrations in robins of different sexes in two regions (Alaska and the Midwest, USA) and evaluated the relationship between blood Pb and robin physiology. We hypothesized that robins would be more exposed to Pb in urban than rural areas, consistent with previous songbird studies (McClelland et al. 2019; Zahor et al. 2024), and predicted that blood Pb concentrations would be higher in more urbanized Indiana than rural Alaska. We also hypothesized that robin exposure to Pb would vary seasonally, as robins consume more earthworms in spring than in fall and winter, when they more heavily rely on fruit (Vanderhoff et al. 2020). We thus predicted that blood Pb should be higher in spring and summer than in fall and winter. Finally, we hypothesized that exposure to Pb would negatively affect robin body condition, and explored any potential relationships between blood Pb and reproductive hormone concentrations. Although Pb is generally known to be an endocrine disruptor (e.g., Herring et al. 2018; He et al. 2020), there is a lack of information on how exposure to Pb impacts the songbird endocrine system, and whether it may be associated with higher or lower reproductive hormone concentrations.

## Materials and methods

### American robin sampling

We collected blood samples from 245 American robins at the Indiana University-Bloomington (IUB) campus and surrounding neighborhoods (*n* = 194, 24 capture locations) as well as at Joint Base Elmendorf-Richardson (JBER) near Anchorage, Alaska, USA (*n* = 51, 18 capture locations). The two study regions are notably different in terms of urbanization, with Bloomington being more urban, with a population of 79,000, whereas JBER, which is located on the outskirts of Anchorage (32,000 people), is more rural. Bloomington has a background soil Pb level of 8.7–239 mg/kg (Urban Environmental Health 2024), whereas JBER has a very low soil Pb burden, less than 10 mg/kg on average (Final Site Characterization Report CG114–22nd Street 2018).

We conducted fieldwork at IUB from September–December 2021 (*n* = 52); January–April (*n* = 46), June (*n* = 8), and September 2022 (*n* = 13); March–May (*n* = 61), September (*n* = 5), and October 2023 (*n* = 9); we also conducted surveys at JBER in May 2023 (*n* = 51). We did not sample robins during July and August because capturing robins during those months is difficult, as robins are less territorial during mid- to late summer in Indiana. We captured robins using polyester mist-nets early in the day using methods described in Jahn et al. (2025a). We banded robins with uniquely numbered metal bands and aged and sexed them using methods described in Pyle (1997). A portion of robins sampled (*n* = 144) could not be sexed in the field, and molecular assessment of sex based on PCR was not successful. We classified robins that hatched in the same calendar year as when we sampled them as hatch year (*n* = 24), and robins hatched in a previous calendar year as after hatch year (*n* = 216). A small subset of robins could not be aged (*n* = 5). We used methods described in Ralph (1993) to measure wing chord (mm) and body mass (g). Before release, we collected ~ 100 µL of blood by puncturing the brachial vein with a sterile insulin needle, which was then transferred to a microcentrifuge tube using a capillary tube (Owen 2011). We stored blood samples whole at -80° C or centrifuged them at 3000 rpm for five minutes, then removed plasma and red blood cells with a pipette and stored them at -80° C. Our analyses included robins with either RBCs (*n* = 133 from Alaska and 82 from Indiana) or whole blood (*n* = 73 from Indiana), and 39 birds with both blood samples (only Indiana).

### Blood Pb analyses

We acid digested whole blood samples by using a modified version of a procedure from the University of California, Santa Cruz Metx Smith Lab. The primary modifications included adjusting quantities to yield a final nitric acid concentration of 2% to meet instrument requirements for Inductively Coupled Plasma Mass Spectrometry analysis, as well as to assay smaller quantities of available sample. Briefly, we transferred 100 µL of blood to an acid-clean centrifuge tube, which was then weighed. We then added 150 µL of concentrated Q-nitric acid (68%) to begin the digestion process, which was then left to rest overnight. The following day, we added 250 µL hydrogen peroxide (30%) and 4.5 mL milliQ water, and then briefly vortexed the sample before again leaving it overnight. We vortexed samples for five minutes, and then removed an aliquot (3 mL) of the supernatant and transferred it to another centrifuge tube.

We re-centrifuged RBC samples and zeroed them out on an analytical balance, added 250 µL of saline, and recorded the weight. We transferred 350 µL of the sample to a tared centrifuge tube, which was weighed and then mixed with milliQ water for a total 5 mL volume. We derived blood volume by dividing blood mass by an average blood density of 1.05. We then calculated dilution factors by dividing the sample volume (5 mL) by blood volume per sample.

We collected lead concentrations for whole blood and RBCs on an Agilent 7700 quadrupole ICP-MS, which is capable of detection limits as low as parts per trillion and which can also measure multiple elements within a single analysis, yielding highly precise results (Haefliger et al. 2020). Prior to sample analysis, we ran a series of calibration standards to provide a calibration curve for the determination of elemental concentrations, with solutions spiked on-line with an internal standard of 25 ng/g indium to correct for instrumental drift. Each sample analysis consisted of five blocks of 100 sweeps, which we then averaged along with relative standard deviation. We interspersed procedural blanks throughout each analytical session to confirm negligible background (Pb consistently < 0.002 ng) and analyzed the USGS standard BCR-2 (*n* = 3) to confirm reproducibility of certified Pb concentrations; BCR-2 Pb recovery was within ± 7% of certified values (11.7 mg/kg, 10.9 mg/kg, and 10.3 mg/kg compared with 11 mg/kg for published values). As part of the data preparation, we corrected results and their respective relative standard deviations using the dilution factor calculated during digestion, then converted to micrograms per deciliter (µg/dL).

### Comparing Pb values between whole blood and RBC samples

We used a set of 17 paired whole blood and RBC samples that were digested at the same time to explicitly compare blood Pb concentrations, given that 84% of birds had only either sample type. We fit a generalized linear model in R with Pb concentrations in RBCs predicting those in whole blood (Gaussian response), detecting a strong, positive relationship (*F*_*1,15*_ = 38.6, *p* < 0.001, *R*^*2*^ = 0.70; Figure S1). We used the estimated intercept (*α* = 1.32) and slope (*β* = 0.40) to predict Pb concentrations in whole blood for any robins where we collected Pb concentrations in only RBCs (*n* = 133 from Alaska, *n* = 82 from Indiana ). We then used these adjusted values in subsequent analyses. For any robins with Pb concentration data from both whole blood and RBCs (*n* = 39), we applied this correction to the RBC data and averaged values from both samples (known whole blood and corrected RBC values were highly correlated; ρ = 0.9, *p* < 0.001).

### Hormone extraction and assays

We extracted testosterone (T; from male robins) and estradiol (E_2_; from female robins) from plasma using 40 µL to assay for T and 100 µL to assay for E_2_. We extracted T by following a standard solid-phase column extraction protocol (Newman et al. 2008), in which we ran 90% methanol through Sep-Pak C18 vacuum columns (Waters Corporation—Part # WAT043395). We extracted E_2_ using a liquid–liquid extraction method by suspending 100 µL plasma in 200 µL ultrapure water and mixing with 1.5 mL diethyl ether. We then flash-froze the aqueous phase in a dry ice bath, and decanted the ether phase. We repeated this procedure three times, after which we evaporated the ether under a gentle stream of air using an evaporation manifold.

We measured extraction efficiency at 68% for the solid-phase extraction method and 86% for the liquid–liquid method, using a four-sample spike series outlined under “Sample Recoveries” of the ELISA Kit Manual: (1) unextracted spike, (2) extracted spike, (3) plasma, and (4) spiked plasma. We subtracted raw plasma values from the spiked plasma values, and compared the average recovery in the spiked plasma to the recovery of identical spikes in the extracted assay buffer. We divided all resulting concentration values by the respective sample recoveries so as to correct for systematic sample loss during extraction.

We measured T using the Testosterone High Sensitivity enzyme-linked immunosorbent assay (ELISA) Kit (Enzo Life Sciences—Catalogue # ADI-900-176) and measured E_2_ using 17β-Estradiol High Sensitivity ELISA Kit (Enzo Life Sciences—Catalogue # ADI-901-174). We validated these to confirm that robin plasma extracts can be measured in absolute terms, with assay validations achieving parallelism and linearity between observed and expected concentrations (T: *R*^2^ = 0.99, β = 1.0; E_2_: *R*^*2*^ = 0.99, β = 0.95).

We measured T and E_2_ concentrations from the plasma extracts using protocols described in the assay kit manuals. We used a BioTek Epoch microplate spectrophotometer to measure absorbance at 405 nm, with the BioTek Gen6 Data Analysis Software recording results. We then transformed sample absorbance to a concentration in pg/mL using a four-point logistic curve, which we based on a standard curve made by diluting a known concentration of each hormone according to the respective kit procedures. We then ran the extracted samples in duplicate (in the same plate) and averaged the data across duplicates for a single measure per sample, randomizing populations across plates. We excluded samples if they exhibited a replicate coefficient of variation (CV) of the optical density (OD) greater than 10%. We also excluded samples if they measured below the sensitivity of the assay (i.e., 2.6 pg/mL for T and 14 pg/mL for E_2_).

We used a pool of robin plasma extract as a control to assess within-plate and between-plate CVs. We ran four T plates and two E_2_ plates. The average within-plate OD CV for T was 4.8%, while the average concentration CV was 9.1%. The between-plate concentration CV for T was 11.4%. The average within-plate OD CV for E_2_ was 2.5%, and the average concentration CV was 4.0%. The between-plate concentration CV for E_2_ was 4.9%.

### Statistical analysis

We used generalized linear mixed models (GLMMs) and generalized additive mixed models (GAMMs) to assess the relationship between regional, seasonal, and demographic factors and blood Pb (log_10_ transformed), using R and either the *lmerTest* or *mgcv* packages (Kuznetsova et al. 2017; Wood 2017). We fit GLMMs and GAMMs using restricted maximum likelihood and a Gaussian response, and all models included a random intercept of capture site. We first used a GLMM to test effects of sex and age, using our full dataset and controlling for ordinal date (*n* = 245). To next assess regional effects, we used another GLMM fit to data from spring (*n* = 162), as Alaska was only sampled in May 2023; this model included region and controlled for sex and ordinal date (age was excluded, as all birds during spring were after hatch year). We then used a GAMM to test for seasonal effects, using only the Indiana data (*n* = 194); this model included year, sex, and age as well as a cyclic smooth term for month. We derived marginal and conditional *R*^*2*^ for GLMMs (and overall *R*^*2*^ for models fit with *mgcv*) using the *performance* package (Lüdecke et al. 2021).

To test the relationships between blood Pb and robin body condition and reproductive hormones, we used three additional GLMMs. Because robins are sexually dimorphic (Vanderhoff et al. 2020), we estimated body condition separately for males, females, and individuals of unknown sex using the residuals of sex-specific linear models that regressed wing chord on body mass (Labocha and Hayes 2012). We then used a GLMM predicting body condition (Gaussian response) as a function of blood Pb while controlling for age, sex, and ordinal date (*n* = 216). Lastly, we fit two separate GLMMs predicting T and E_2_ (Tweedie response) as functions of blood Pb while controlling for region and ordinal date; all analyzed reproductive hormone data were from 2023 and included robins of known and unknown sex (T = 62 individuals, E_2_ = 41 individuals). We again derived marginal and conditional *R*^*2*^ for GLMMs, and overall *R*^*2*^ for models fit using *mgcv*, using the *performance* package (Lüdecke et al. 2021).

## Results

Across our sample of 245 robins in IN and AK, blood Pb concentrations ranged across two orders of magnitude, from 0.8 to 30.9 µg/dL (geometric mean = 3.4 ± 1.0 SE). Of the 13 robins with blood Pb above 10 µg/dL, all but one were from Indiana and most were after hatch year birds (69%); however, these individuals were evenly distributed across years, seasons, and sexes.

**Fig. 1:**
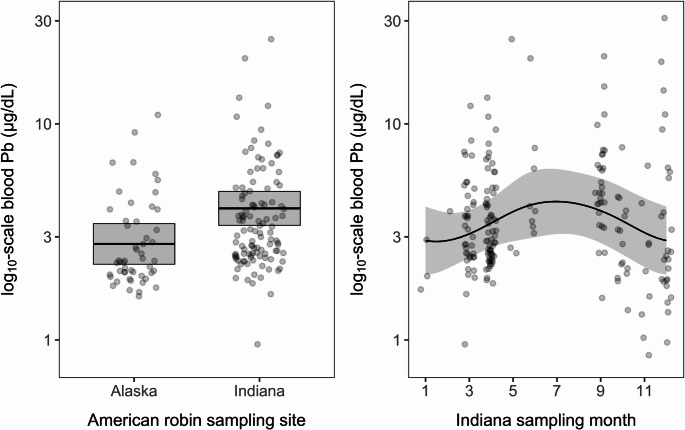
Relationships between region, season, and blood Pb concentrations. Plots show the fitted values and 95% CIs from the GLMM (left panel, spring data only) or GAMM (right panel, Indiana only) alongside raw data (jittered to reduce overlap). The Y axes are shown on the log_10_ scale

When assessing the effects of demographic factors on blood Pb across our whole dataset, we identified no effects of sex (χ² = 3.88, *p* = 0.14) or age (χ² = 4.41, *p* = 0.11); the fixed effects explained only 3% of the variation in blood Pb, with more variance accounted for by site effects (*R*^*2*^_*c*_ = 0.13; Table S1). When we focused our analyses on regional factors using our spring data (*n* = 162), we identified strong effects of geography (χ² = 7.47, *p* < 0.01). Birds from Indiana had significantly higher blood Pb (x̄ = 4.06, 95% CI = 3.39–4.87 µg/dL) than birds from Alaska (x̄ = 2.78, 95% CI = 2.24–3.45 µg/dL; Fig. 1). Other factors had no effect (Table S2), with fixed and random effects explaining 9.5% and 16.8% of the variation in blood Pb, respectively (Table S2). Lastly, when we focused our analyses on seasonal factors using our Indiana data (*n* = 194), we identified significant seasonal effects (*F*_*1.5,3*_ = 3.00, *p* = 0.03) but no effects of year, sex, or age (Table S3). Seasonality explained 11.9% of the variation in Indiana blood Pb, for which levels were greatest in spring and summer before declining in fall and winter (Fig. 1).

**Fig. 2:**
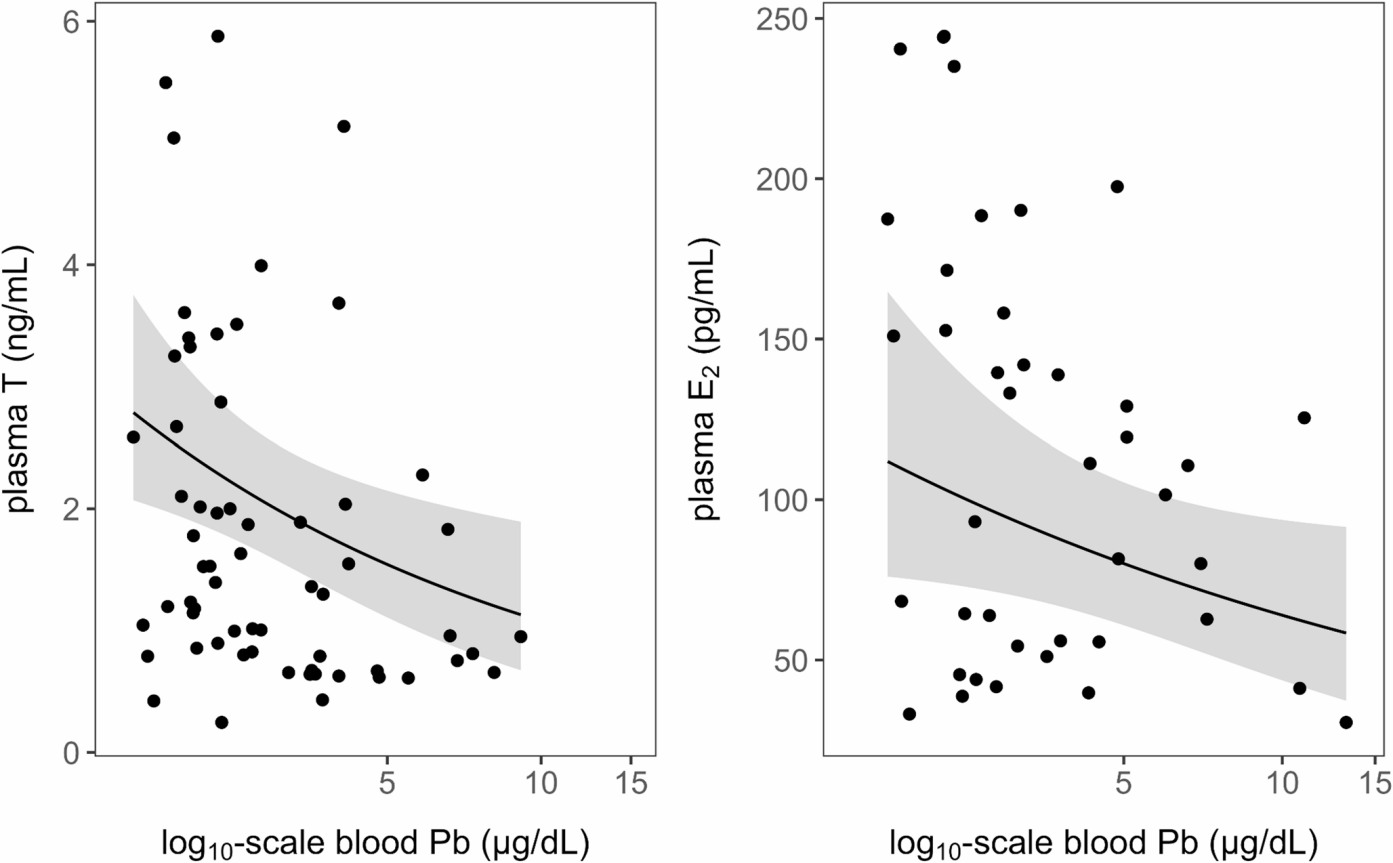
Relationships between blood Pb concentrations and testosterone in males (left) and estradiol in females (right). Plots show the fitted values and 95% CIs from GLMMs (Tweedie response) alongside raw data. The X axes are shown on the log_10_ scale

When assessing potential physiological impacts of Pb, we found no effect of blood Pb on body condition (Table S4). However, our GLMMs of reproductive hormones showed that blood Pb had significant, negative relationships with T for males (β = -1.19, *t* = -2.72, *p* < 0.01) and E_2_ for females (β = -0.75, *t* = -2.00, *p* = 0.05), after adjusting for region and ordinal date (Fig. 2). These GLMs explained 16.9% and 33.9% of the variance in reproductive hormones, respectively.

## Discussion

Our results demonstrate that Pb exposure of American robins is region-specific and seasonal, potentially related to levels of urbanization and shifts in diet, and may have subsequent repercussions on the endocrine system. Our results also suggest that robins are more exposed to soil Pb in more urban vs. more rural areas, and our multi-season sampling in Indiana revealed that exposure of robins to Pb is higher in spring and summer than during the rest of the year. The latter finding is consistent with our hypothesis that the earthworm-heavy spring diet of robins will yield higher Pb exposure, particularly for more Pb-contaminated urban soils. We also detected a negative relationship between Pb concentrations and reproductive hormone concentrations.

The significantly higher blood Pb concentration in spring and summer that we detected in robins is likely driven by the heavy reliance on earthworms at this time of year (Vanderhoff et al. 2020). Additionally, environmental acidification may occur as a result of higher summer temperatures, solar radiation, and humidity, leading to increased Pb bioavailability (Levin et al. 2020). Monitoring seasonal fluctuations in bird blood Pb may thus serve as an indirect method of estimating Pb soil contamination in urban areas. Other species, such as the House sparrow (*Passer domesticus*), have successfully been used as sentinels of human health risks, serving as a proxy for child blood Pb down to the neighborhood scale (0.28 km^2^) (Gillings et al. 2024). Robins could fill a similar role, given their ubiquity in anthropogenic environments. Further research on regional and seasonal diet variability and correlation with blood Pb concentrations would shed more light on the seasonal exposure of robins to Pb.

Our results suggest that robin body condition is not significantly impacted by exposure to Pb, regardless of the level of urbanization where they are sampled or the season of sampling. Likewise, previous research on Northern mockingbirds (*Mimus polyglottos*) showed no significant differences in body condition between those sampled at sites with different levels of soil Pb concentrations (McClelland et al. 2019). Nevertheless, body condition in robins and other songbirds is a product of various drivers, such as preparation for breeding in spring and feather molt in late summer (Labocha and Hayes 2012). We also cannot rule out that the seasonal blood Pb concentrations and trends we detected are a product of other factors, such as differences in soil type, habitat, and diet that may differ between Alaska and Indiana, as well as incomplete sampling of both sexes across the year. Robins in our study populations are migratory, with Alaska robins overwintering in the Great Plains region (Jahn et al. 2019) and robins in Indiana being partially migratory, with some remaining in Indiana year-round and others migrating south to the southeastern USA (Jahn et al. 2025b). Thus, evaluating Pb exposure by robins across their breeding and non-breeding range could uncover potential region-specific sources of exposure.

American robins exposed to higher levels of Pb exhibited lower testosterone and estradiol concentrations, suggesting that Pb impacts the avian endocrine system. However, further experimental research is necessary to support this and to understand potential mechanisms, since sex hormone levels in birds may be impacted by habitat, diet, season, and migratory condition (reviewed by Ketterson and Grieves 2025). Medullary bone is a specialized tissue developed during the breeding season to act as a calcium reservoir for producing egg shells (Canoville et al. 2019) and it is well established that Pb substitutes for calcium during bone formation if present in the blood (Pounds et al. 1991; Florea et al. 2013). Therefore, one explanation for the negative relationship between estradiol and blood Pb is that, as female birds experience increasing estradiol levels as they prepare to breed, they fix more calcium and thus Pb into their medullary bone, such that their blood Pb concentrations decrease. The opposite is well established during pregnancy in humans (Téllez-Rojo et al. 2004). A strong test of this hypothesis could include a study where birds are dosed with Pb over a sufficiently long duration, hormone and blood Pb concentrations are monitored simultaneously, and medullary bone samples are tested for Pb. A negative correlation between estradiol and blood Pb and a positive correlation between estradiol and medullary bone Pb would provide support for the hypothesized mechanism. Although the effects of Pb exposure on male avian endocrine function remain poorly understood, a study on Japanese Quail (*Coturnix japonica*) suggested that ingestion of high levels of Pb (at least 500 ppm) results in disruption of the hypothalamus-pituitary-gonadal axis and testis atrophy (Zheng et al. 2023). Although the specific mechanisms remain unknown in robins, it is possible that the negative relationship between blood Pb and testosterone that we observed could result from similar mechanisms as those found in Japanese Quail.

A potentially fruitful line of future research with robins as bioindicator models is to examine the impacts of urbanization on the avian endocrine system, as elevated Pb levels are widely associated with urban environments (e.g., Filippelli et al. 2024) and because there is currently a lack of consensus on the impacts of urbanization on the avian endocrine system (reviewed by Deviche et al. 2023). Results of such research likely depend on the type of hormone in question and the dietary ecology of the study organism. House sparrows exhibit a positive relationship between urbanization, Pb, and corticosterone (White et al. 2022). In terms of reproductive hormones, adult European starlings (*Sturnus vulgaris*) show no significant relationship between feather Pb and testosterone concentrations (Ross et al. 2024), and testosterone concentrations of male tree sparrows (*Passer montanus*) are not significantly different between sites with different levels of soil Pb (Yang et al. 2020).

More generally, our results support findings of previous research suggesting that robins and other songbirds can serve as effective biomonitors of soil Pb contamination (e.g., Monzalvo-Santos et al. 2016; Zahor et al. 2024). Robins are relatively easily captured using mist-nets, their large size allows drawing larger blood volumes relative to many other passerines, and they inhabit a variety of habitats across North America (Scheifler et al. 2006; Biswas 2023; Gillings et al. 2024; Zahor et al. 2024). Nevertheless, the use of birds as bioindicators of heavy metals requires further advancement in detection and monitoring techniques (; French et al. 2017; Herring et al. 2018; Center for Devices and Radiological Health 2022). Currently, anodic stripping voltammetry devices are the standard for testing blood Pb concentrations in wildlife, but this leaves a gap in low-level detection, with a detection limit of only 3.3 µg/dL (Zahor et al. 2024). Previously, studies referenced background levels as being less than 20 µg/dL, but lower Pb concentrations have been cited more recently as being associated with subclinical poisoning (e.g., Herring et al. 2020). One study on Tasmanian wedge-tailed eagles (*Aquila audax fleayi*) highlights the need for low detection limits, with a geometric mean of only 3.08 µg/dL in nestlings and 1.03 µg/dL for all the individuals sampled (Pay et al. 2021). Andean condors (*Vultur gryphus*) have a mean blood Pb of 15.47 µg/dL, and half of common ravens (*Corvus corvax*) sampled had blood Pb greater than 10 µg/dL (Pain et al. 2019).

In terms of linking risk of exposure to heavy metals to movement and behavior, miniaturized biologging technologies now exist that allow tracking the movement and activity levels of songbirds at fine spatial and temporal scales (reviewed by Flack et al. 2022). Such devices could be applied in the context of heavy metals to understand where and when birds are exposed to Pb as well as how Pb exposure affects movement decisions and population dynamics. Given the rapid declines in the abundance of various North American bird taxa during the last 50 years (Rosenberg et al. 2019), future research on songbird exposure to heavy metals has the potential to unveil novel behavioral and health impacts from exposure to heavy metals and whether these could be a hidden driver of population change.

## Supplementary Information

Below is the link to the electronic supplementary material.

Supplementary Material 1

## Data Availability

Data will be made publicly available in the Dryad database.
