# Supplementary material for: Region- and season-specific exposure to lead in a common North American songbird
Source: Ecotoxicology. 2026 Mar 27;35(4):90. doi: 10.1007/s10646-026-03070-3 (PMC13021721; doi:10.1007/s10646-026-03070-3)
Supplement: Supplementary file 1 — Supplementary Material 1 [file 10646_2026_3070_MOESM1_ESM.docx]

Figure S1. Relationship between Pb concentrations in 17 paired whole blood and RBC samples used to adjust our larger sample of RBC-derived American Robin blood Pb data. The darker band and line show the 95% confidence interval and fitted values for our GLM, whereas the lighter band and line show predictions from this model extended to the full range of our RBC-derived blood Pb data. The dashed line shows the 1:1 relationship between blood Pb concentrations.


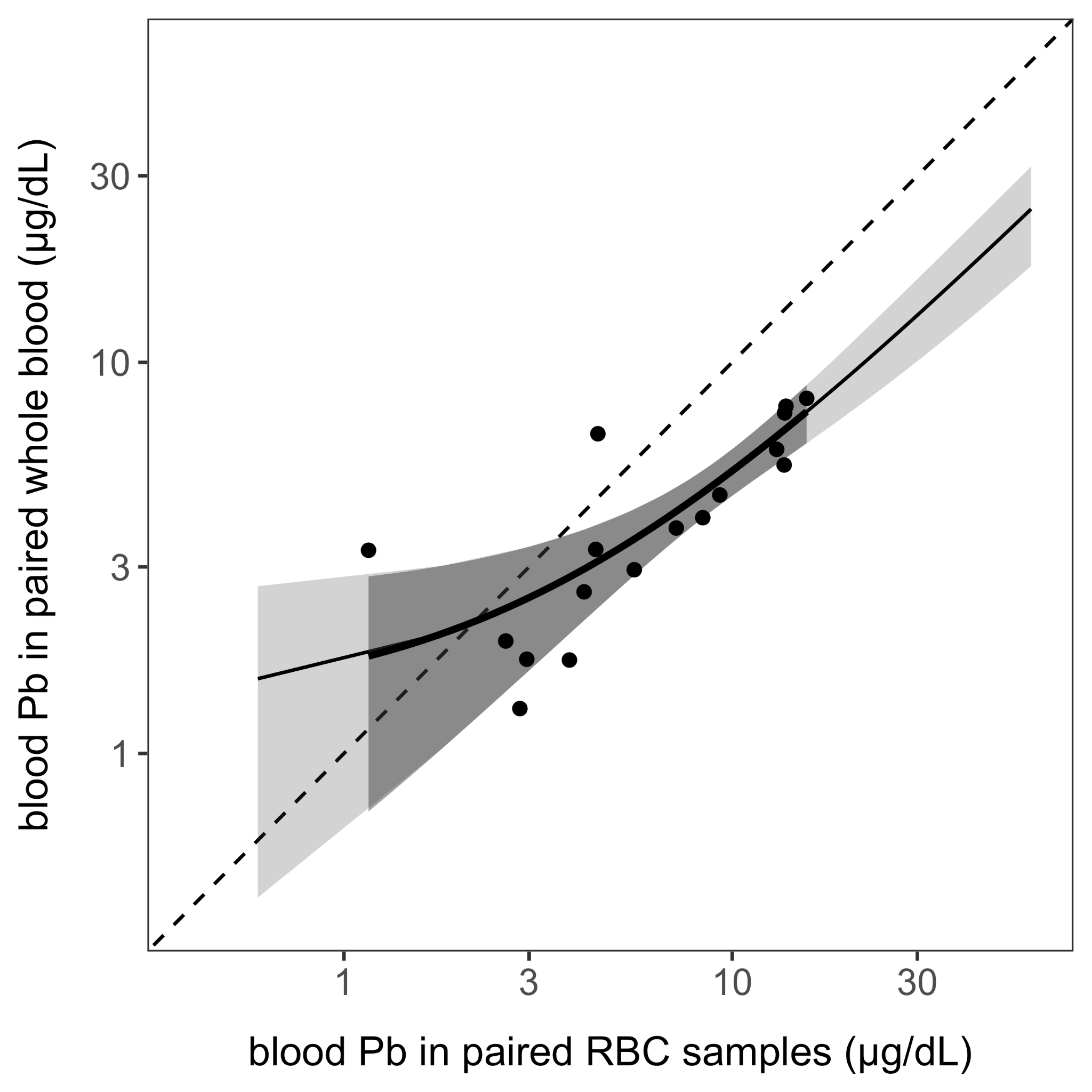


Table S1. GLMM results from the analysis of demographic factors predicting log_10_-transformed American Robin blood Pb concentration in our whole dataset (*n* = 245).

| **Coefficient** | **β** | **SE** | ***t*** | ***p*** |
| --- | --- | --- | --- | --- |
| Intercept | 0.76 | 0.10 | 7.80 | < 0.001 |
| Male | -0.11 | 0.06 | -1.84 | 0.07 |
| Unknown sex | -0.12 | 0.06 | -1.89 | 0.06 |
| AHY | -0.13 | 0.06 | -2.07 | 0.04 |
| Unknown age | -0.13 | 0.13 | -1.03 | 0.31 |
| Ordinal date | 0.00 | 0.00 | -0.39 | 0.70 |

Table S2. GLMM results from the analysis of geographic factors predicting log_10_-transformed American Robin blood Pb concentration in our spring data subset (*n* = 162).

| **Coefficient** | **β** | **SE** | ***t*** | ***p*** |
| --- | --- | --- | --- | --- |
| Intercept | 0.35 | 0.15 | 2.29 | 0.02 |
| Indiana | 0.17 | 0.06 | 2.73 | 0.01 |
| Male | -0.10 | 0.05 | -1.97 | 0.05 |
| Unknown sex | -0.10 | 0.06 | -1.74 | 0.08 |
| Ordinal date | 0.001 | 0.001 | 1.44 | 0.15 |

Table S3. GAMM results from the season-focused analysis of log_10_-transformed American Robin blood Pb concentration within our Indiana subset of the data (*n* = 194). The table reports the parametric results.

| **Coefficient** | **β** | **SE** | ***t*** | ***p*** |
| --- | --- | --- | --- | --- |
| Intercept | 0.66 | 0.11 | 6.22 | < 0.001 |
| 2022 | 0.07 | 0.07 | 1.05 | 0.29 |
| 2023 | 0.07 | 0.08 | 0.85 | 0.40 |
| Male | -0.06 | 0.08 | -0.77 | 0.45 |
| Unknown sex | -0.06 | 0.08 | -0.65 | 0.52 |
| AHY | -0.12 | 0.06 | -1.83 | 0.07 |
| Unknown age | -0.08 | 0.13 | -0.59 | 0.56 |

Table S4. GLMM results from American Robin blood Pb concentration predicting body condition (*n* = 216).

| **Coefficient** | **β** | **SE** | ***t*** | ***p*** |
| --- | --- | --- | --- | --- |
| Intercept | -2.48 | 2.08 | -1.19 | 0.23 |
| log_10_ Pb | -0.78 | 1.16 | -0.67 | 0.50 |
| Male | 0.01 | 1.35 | 0.01 | 1.00 |
| Unknown sex | -0.40 | 1.34 | -0.30 | 0.76 |
| AHY | 1.69 | 1.13 | 1.50 | 0.14 |
| Unknown age | 2.84 | 2.37 | 1.20 | 0.23 |
| Ordinal date | 0.01 | 0.004 | 2.45 | 0.02 |
